# COVID-19 vaccine hesitancy is associated with beliefs on the origin of the novel coronavirus in the UK and Turkey

**DOI:** 10.1017/S0033291720004067

**Published:** 2020-10-19

**Authors:** Gul Deniz Salali, Mete Sefa Uysal

**Affiliations:** 1 Department of Anthropology, University College London, London WC1H 0BW, UK; 2 Department of Psychology, Dokuz Eylul University, Izmir, Turkey

**Keywords:** Cognitive bias, conspiracy beliefs, COVID-19, cultural evolution, health communication, public health, vaccine hesitancy

## Abstract

**Background:**

Much research effort is focused on developing an effective vaccine for combatting coronavirus disease 2019 (COVID-19). Vaccine development itself, however, will not be enough given that a sufficient amount of people will need to be vaccinated for widespread immunity. Vaccine hesitancy is on the rise, varies across countries, and is associated with conspiratorial worldview. Given the rise in COVID-19-related conspiracy theories, we aimed to examine the levels of COVID-19 vaccine hesitancy and its association with beliefs on the origin of the novel coronavirus in a cross-cultural study.

**Methods:**

We conducted an online survey in the UK (*N* = 1088) and Turkey (*N* = 3936), and gathered information on participants' willingness to vaccinate for a potential COVID-19 vaccine, beliefs on the origin of the novel coronavirus, and several behavioural and demographic predictors (such as anxiety, risk perception, government satisfaction levels) that influence vaccination and origin beliefs.

**Results:**

In all, 31% of the participants in Turkey and 14% in the UK were unsure about getting themselves vaccinated for a COVID-19 vaccine. In both countries, 3% of the participants rejected to be vaccinated. Also, 54% of the participants in Turkey and 63% in the UK believed in the natural origin of the novel coronavirus. Believing in the natural origin significantly increased the odds of COVID-19 vaccine acceptance.

**Conclusions:**

Our results point at a concerning level of COVID-19 vaccine hesitancy, especially in Turkey, and suggest that wider communication of the scientific consensus on the origin of the novel coronavirus with the public may help future campaigns targeting COVID-19 vaccine hesitancy.

Much research effort is focused on developing an effective vaccine for combatting coronavirus disease 2019 (COVID-19). Vaccine development itself, however, will not be enough given that a sufficient amount of people will need to be vaccinated for widespread immunity. Vaccine hesitancy is on the rise, varies across countries, and is associated with conspiratorial worldview (Gallup, 2019; Hornsey, Harris, & Fielding, 2018). Given the rise in COVID-19-related conspiracy theories (Freeman et al., 2020), we aimed to investigate the determinants of, and association between COVID-19 vaccine hesitancy and the beliefs on the origin of the novel coronavirus in a cross-cultural study.

We conducted an anonymous online survey throughout May 2020 in the UK (*n* *=* 1088) and Turkey (*n* *=* 3936), and gathered information on participants' willingness to vaccinate for a potential COVID-19 vaccine (yes / no / not sure) and beliefs on the origin of the virus (natural/artificial/not sure). All participants were above 18, residing either in the UK or Turkey. Detailed description and summary statistics of the survey variables are available on the Open Science Framework website (https://osf.io/3gz5a/).

COVID-19 vaccine hesitancy was higher in Turkey: 31% of the participants in Turkey and 14% in the UK were unsure about getting themselves and, if they have, their children vaccinated (*n* = 5024, χ^2^ = 99.5, *p* *<* 0.001). In both countries, 3% of the participants rejected to be vaccinated. More participants in the UK believed in the natural origin of the virus (54% in Turkey, 63% in the UK, *n* = 5024, χ^2^ = 24.6, *p* *<* 0.001), and 18% in Turkey and 12% in the UK thought the origin to be artificial, i.e. human-made. COVID-19 vaccine acceptance rates were higher among the participants who believed in the natural origin (Fig. 1, proportion tests *p* *<* 0.001 for both countries). We conducted logistic regression models to investigate factors that affected the odds of (i) COVID-19 vaccine acceptance, (ii) believing in the natural origin of the virus (for model tables see https://osf.io/3gz5a/). Odds of vaccine acceptance were 26% higher in Turkey and 63% higher in the UK if a person believed in the natural origin, compared to those who were not sure about the origin (*p* *<* 0.001).

**Fig. 1.**
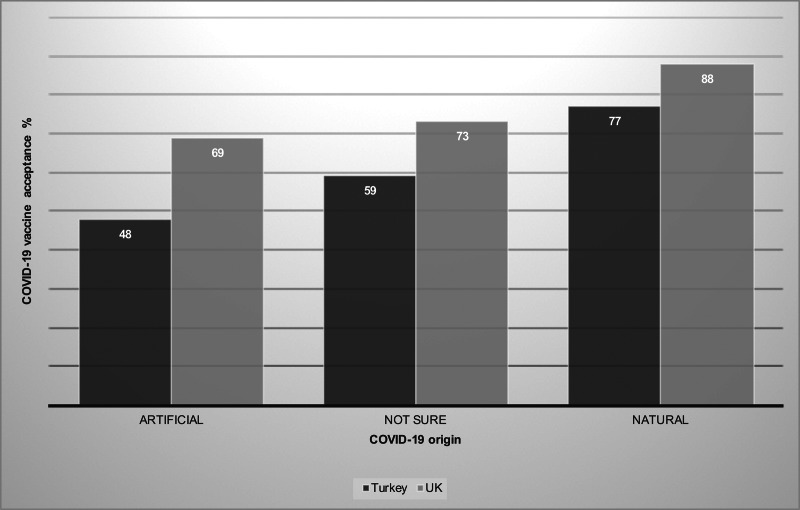
Percentage of participants who responded ‘yes’ to the question of whether they would vaccinate themselves and their children for COVID-19 in Turkey (*n* *=* 3936) and the UK (*n* *=* 1088) based on their belief on the origin of the coronavirus (artificial, not sure, natural).

Several other behavioural and demographic factors influenced vaccination and origin beliefs. Participants who had higher COVID-19-related anxiety scores had higher odds of vaccine acceptance (Turkey: OR 1.48, 95% CI 1.32–1.65, *p* *<* 0.001; UK: OR 1.36, 95% CI 1.04–1.77, *p* *<* 0.05), which can be explained by the adaptive function of anxiety in decreasing mortality risk (Nesse, 2005). Perceived risk of catching COVID-19 (Turkey: OR 1.03, 95% CI 1–1.06, *p* *=* 0.06; UK: OR 1.12, 95% CI 1.04–1.2, *p* *<* 0.01) and frequency of watching/listening/reading to the news had positive effects on vaccine acceptance (Turkey: OR 1.06, 95% CI 1–1.11, *p* *<* *0.05*; UK: OR 1.24, 95% CI 1.05–1.47, *p* *<* 0.05). The degree of satisfaction with government's response to the pandemic was negatively associated with the belief in the natural origin (Turkey: OR 0.77, 95% CI 0.72–0.83, *p* *<* 0.001; UK: OR 0.77, 95% CI 0.67–0.88, *p* *<* 0.001), but not vaccine acceptance.

Compared to women, men in Turkey were more likely to accept a COVID-19 vaccine (Turkey: OR 1.47, 95% CI 1.26–1.71; *p* *<* 0.001; UK: OR 1.44, 95% CI 0.99–2.1, *p* *=* 0.06), and believe in the natural origin of the virus (Turkey: OR 1.23, 95% CI 1.07–1.41, *p* *<* 0.01; UK: OR 1.26, 95% CI 0.94–1.68, *p* *=* 0.13). Having a graduate degree and children decreased the odds of vaccine acceptance in Turkey, but not in the UK (Turkey: graduate/non-graduate degree OR 0.69, 95% CI 0.58–0.81, *p* *<* 0.001; children/no children OR 0.82, 95% CI 0.69–0.96, *p* *<* 0.05). These two factors were significantly associated with the origin beliefs in both countries. Participants without any children were 41% more likely in Turkey, and 85% in the UK to believe in the natural origin of the virus (*p* *<* 0.001). Participants with postgraduate degrees had increased odds of believing in the natural origin compared to those without a graduate degree (Turkey: OR 1.63, 95% CI 1.31–2.03, *p* *<* 0.001, UK: OR 2.40, 95% CI 1.70–3.39, *p* *<* 0.001). UK participants who reported their ethnicity as ‘white’ were twice more likely to believe in the natural origin compared to the other ethnicities (*p* *<* 0.001).

Some country-level differences might have contributed to the observed differences in the origin beliefs and vaccine hesitancy. Participants in Turkey reported a lower mean perceived life expectancy (perceived probability of living up to 75 or more from a scale of 0–100: 75 in the UK, 57 in Turkey). Individuals in countries with lower life expectancy (i.e. increased mortality risk) may exhibit increased threat perception and out-group mistrust, promoting a conspiratorial worldview. Moreover, the mean financial satisfaction score was lower in Turkey (from a scale of 0–100: 67 in the UK, 48 in Turkey). Financial satisfaction was indeed a significant predictor explaining the origin beliefs in both countries (Turkey: OR 1.05, 95% CI 1.02–1.07, *p* *<* 0.001; UK: OR 1.07, 95%, CI 1.01–1.13, *p* *<* 0.05). Women in Turkey were more hesitant about a COVID-19 vaccine, consistent with a previous study reporting high levels of vaccine hesitancy among young, educated mothers (Özceylan, Toprak, & Esen, 2020). Because women are more likely to take healthcare decisions for their children, they may also be more likely to seek out information about vaccines and be exposed to online anti-vaccination content (Smith & Graham, 2019). Moreover, women score higher on disgust sensitivity (Haidt, McCauley, & Rozin, 1994), which is associated with vaccine hesitancy (Hornsey et al., 2018; Miton & Mercier, 2015).

The observed association between virus origin beliefs and COVID-19 vaccine hesitancy may be rooted in our evolved cognitive biases. Some beliefs, for example, spread faster because they are more in line with our intuitions (intuitive bias), and hence easier to understand and remember. Scientific information, such as vaccines are safe, are often not intuitive, making them harder to be disseminated (Boudry, Blancke, & Pigliucci, 2015; Miton & Mercier, 2015). Besides, the consensus on the natural origin of the novel coronavirus among scientists (Calisher et al., 2020) may not be as attractive a belief as to the one that the virus was originated in a laboratory in Wuhan. The presence of a biological laboratory in the same town where the virus has spread from makes the laboratory-origin belief much more attractive to our minds that have evolved to recognize patterns. Our results point at a concerning level of COVID-19 vaccine hesitancy, especially in Turkey, and suggest that wider communication of the scientific consensus on the origin of the novel coronavirus with the public may help future campaigns targeting COVID-19 vaccine hesitancy.
